# Refined innate plasma signature after rVSVΔG-ZEBOV-GP immunization is shared among adult cohorts in Europe and North America

**DOI:** 10.3389/fimmu.2023.1279003

**Published:** 2024-01-03

**Authors:** Paola Andrea Martinez-Murillo, Angela Huttner, Sylvain Lemeille, Donata Medaglini, Tom H. M. Ottenhoff, Ali M. Harandi, Arnaud M. Didierlaurent, Claire-Anne Siegrist

**Affiliations:** ^1^ Center of Vaccinology, Department of Pathology and Immunology, Faculty of Medicine, University of Geneva, Geneva, Switzerland; ^2^ Center for Vaccinology, Geneva University Hospitals, Geneva, Switzerland; ^3^ Division of Infectious Diseases, Geneva University Hospitals, Geneva, Switzerland; ^4^ Faculty of Medicine, University of Geneva, Geneva, Switzerland; ^5^ Center for Clinical Research, Geneva University Hospitals, Geneva, Switzerland; ^6^ Laboratory of Molecular Microbiology and Biotechnology, Department of Medical Biotechnologies, University of Siena, Siena, Italy; ^7^ Department of Infectious Diseases, Leiden University Medical Center, Leiden, Netherlands; ^8^ Department of Microbiology and Immunology, Sahlgrenska Academy, University of Gothenburg, Gothenburg, Sweden; ^9^ Vaccine Evaluation Centre, BC Children’s Hospital Research Institute, University of British Columbia, Vancouver, BC, Canada

**Keywords:** innate plasma signature, rVSVΔG-ZEBOV-GP, biomarkers, adverse events, immunogenicity

## Abstract

**Background:**

During the last decade Ebola virus has caused several outbreaks in Africa. The recombinant vesicular stomatitis virus-vectored Zaire Ebola (rVSVΔG-ZEBOV-GP) vaccine has proved safe and immunogenic but is reactogenic. We previously identified the first innate plasma signature response after vaccination in Geneva as composed of five monocyte-related biomarkers peaking at day 1 post-immunization that correlates with adverse events, biological outcomes (haematological changes and viremia) and antibody titers. In this follow-up study, we sought to identify additional biomarkers in the same Geneva cohort and validate those identified markers in a US cohort.

**Methods:**

Additional biomarkers were identified using multiplexed protein biomarker platform O-link and confirmed by Luminex. Principal component analysis (PCA) evaluated if these markers could explain a higher variability of the vaccine response (and thereby refined the initial signature). Multivariable and linear regression models evaluated the correlations of the main components with adverse events, biological outcomes, and antibody titers. External validation of the refined signature was conducted in a second cohort of US vaccinees (n=142).

**Results:**

Eleven additional biomarkers peaked at day 1 post-immunization: MCP2, MCP3, MCP4, CXCL10, OSM, CX3CL1, MCSF, CXCL11, TRAIL, RANKL and IL15. PCA analysis retained three principal components (PC) that accounted for 79% of the vaccine response variability. PC1 and PC2 were very robust and had different biomarkers that contributed to their variability. PC1 better discriminated different doses, better defined the risk of fever and myalgia, while PC2 better defined the risk of headache. We also found new biomarkers that correlated with reactogenicity, including transient arthritis (MCP-2, CXCL10, CXCL11, CX3CL1, MCSF, IL-15, OSM). Several innate biomarkers are associated with antibody levels one and six months after vaccination. Refined PC1 correlated strongly in both data sets (Geneva: r = 0.97, P < 0.001; US: r = 0.99, P< 0.001).

**Conclusion:**

Eleven additional biomarkers refined the previously found 5-biomarker Geneva signature. The refined signature better discriminated between different doses, was strongly associated with the risk of adverse events and with antibody responses and was validated in a separate cohort.

## Introduction

Since the identification of the ebolaviruses in 1976, several outbreaks of Ebola disease have been identified in sub-Saharan Africa. Ebola virus disease (EVD) induces a high mortality rate (50-90%) and can result in uncontrolled epidemics, as witnessed in 2014-16 during the largest Ebola outbreak ever reported (1). The international response to this outbreak supported international collaborations to test EVD vaccine candidates. rVSVΔG-ZEBOV-GP, the most advanced candidate at that time, is a live-attenuated vaccine whose vesicular stomatitis virus glycoprotein-encoding gene has been deleted (VSVΔG) and replaced with the Zaire Ebola virus (ZEBOV-GP) glycoprotein. This vaccine induced 100% protection against EVD in challenged non-human primates (NHP) (2–4).

rVSVΔG-ZEBOV-GP proved safe and immunogenic in different clinical trials held in the USA, Europe and Africa (5–11), but induces transient reactogenicity (12). It was shown to be effective within days in the ring vaccination trial held in 2015 in Guinea (10) and during the 2018–19 outbreak in the Democratic Republic of Congo (13). All these findings supported fast tracked vaccine licensure, resulting in a prequalification by WHO for rVSVΔG-ZEBOV-GP to be used in countries at high risk in 2019 (14), and to its license under the name of Ervebo^®^ by the FDA (15) and by the EMA (16).

Although rVSVΔG-ZEBOV-GP is highly effective against EVD, only a few studies have explored its principal innate and adaptive induced immune mechanisms and its ability to induce early protection. Studies in NHP models have demonstrated that antibodies and CD4^+^ T-cells are necessary for rVSV-EBOV-mediated protection against lethal infection, while CD8^+^ T-cells play a minor role (17). Interestingly, rVSVΔG-ZEBOV-GP induced partial and total protection in NHP as early as 3 and 7 days after challenge, in absence of detectable antigen-specific IgG and low IgM-specific serum antibodies (18), suggesting a role of innate responses in mediating early protection.

rVSVΔG-ZEBOV-GP induces a robust innate immune response characterized by the mobilization of monocytes and natural killer (NK) cell in humans, and NK cell activation and CXCL10 levels correlates with antigen-specific antibody responses (8, 19). Similarly, other rVSV-based vaccines evaluated in NHPs induce the secretion of cytokines/chemokines and NK cell activation [VSV-MARV (20, 21)] and the transcription of genes involved in NK and innate immune pathways [rVSVΔG-LASV-GPC (22)]. We showed in Geneva vaccinees that this mobilization and activation of circulating NK cells was rapid and dose-dependent (23). We also identified the first innate plasma signature response to rVSVΔG-ZEBOV-GP in healthy vaccinees, derived in a European cohort (Geneva, Switzerland) and validated in an African cohort (Lambaréné, Gabón) (24). Among the six monocyte-related cytokines/chemokines which peaked at day 1 post-immunization, five (MCP-1, IL-1Ra, TNF-α, IL-10 and IL-6) defined a signature that was vaccine dose-dependent and correlated with viremia, biological outcomes and adverse events, including transient arthritis (24). Here, we aimed to identify additional markers in Geneva vaccinees that could refine the previous signature and to validate this refined signature in a US cohort.

## Methods

### Study design, population, and key previous outcomes

We used plasma samples obtained from two clinical trials conducted in Europe (phase 1/2, randomized, double-blind, placebo-controlled, dose-finding trial in Geneva, Switzerland [November 2014, to January 2015; NCT02287480]) (12) and in North America (phase 1b, randomized, double-blind, placebo-controlled, dose-response trial in the USA [Dec 5, 2014, to June 23, 2015; NCT02314923]) (25). The trial protocols were reviewed and approved by the WHO’s Ethics Committee as well as by local ethics committees (USA trial: the Chesapeake Institutional Review Boards (Columbia, MD, USA) and the Crescent City Institutional Review Board (New Orleans, LA, USA); Geneva trial: the Geneva Cantonal Ethics Commission and the Swiss Agency for Therapeutic Products (Swissmedic). All participants had provided written informed consent to participate in those studies (12, 25).

As genetic and environmental factors may influence vaccine response, we used the Geneva trial as the derivation cohort (n=115) and the US trial as the validation cohort (see **Supplementary Figure 1**). As a wider range of vaccine doses were tested in this US trial (7, 9), we randomly choose a subset of individuals (n=130) grouped to best match Geneva low dose (n=48), high dose (n=60) and placebo (n=22) recipients (**Supplementary Figure 1**).

### Pilot high-throughput screening in plasma from Geneva vaccinees

O-link (OLINK AB, Uppsala) is a semi-quantitative assay based on Proximity Extension Assay (PEA) technology with no cross reactivity. It measures proteins via an antibody-mediated detection system linked to synthetic DNA. The method has been described previously (26). Briefly, paired oligonucleotide-coupled antibodies with overlapping sequences are allowed to bind to proteins in the sample. When paired antibodies are brought in proximity to one another through binding to their target, their oligonucleotide sequences overlap to form a PCR target, which can be semi-quantified with real-time PCR. We used three O-link panels (inflammation, immune and metabolic panels, each panel detecting 92 proteins) to screen for 276 markers. Inflammatory panel was tested first, and we evaluated days 0, 1, 3 and 7. Immune and metabolism panels were used later, and we evaluated only day 0 and 1. Following data pre-processing, including quality control, the relative level (NPX) of each of the 276 proteins was assessed. Proteins with more than 30% of samples with NPX values below the limit of detection (n=53) were excluded from further analysis.

**Figure 1:**
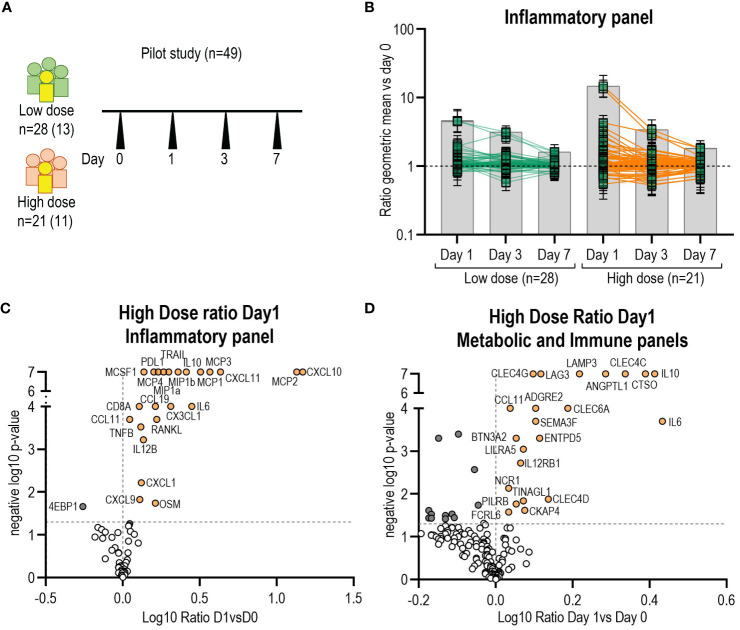
Identification of additional biomarkers by O-link. **(A)** Schematic of the pilot study samples used to screen for new markers (n=49). In yellow and in parenthesis number of participants with arthritis. **(B)** Kinetics of biomarkers from O-link inflammatory panel (96 markers) expressed as the ratio of the mean at day1, day3 and day7 versus day 0. Each square represents the mean for a single marker and confidence interval is included. Volcano plots from O-link inflammatory **(C)** and metabolic panels **(D)** of the high dose group displaying the log10 fold change (x axis) against the t test-derived negative log10 statistical P value (y axis) for all proteins differentially secreted between day1 and day0. Thresholds (dotted grey line), p-value cut-off was fixed at 0.05 (1,3 negative log10) and fold change cut-offs was 1 (0 in the log10 scale). P-value of zero was set up as 0,0000001 (7 neg log10). Open circles represent all proteins below the p-value and in dark grey all proteins below fold change cut-offs. Proteins above the fold-change cut off are labelled as orange circles.

In this pilot screening, we selected a subgroup of participants of the Geneva cohort (n=49), including all participants that reported transient arthritis and matched the samples by dose, sex and age (**Figure 1A**), with the aim to identify potential arthritis-associated biomarkers. We first assessed the number of markers peaking at D1, D3 and D7 (**Figure 1B**). Subsequently, the identified biomarkers were confirmed and quantified by Luminex in each participant of the Geneva cohort (n=115).

### Quantification of biomarkers by Luminex assay

A customized Luminex assay (Magnetic Luminex assay, R&D Systems) was used to measure the plasma concentration of most of the markers identified by O-link, as some were not available for testing with the Luminex technology. Assays were performed according to the supplier’s instructions using) the Luminex xMAP Technology (Luminex Corporation) and read on the Bio-Plex 200 array reader (Bio-Rad Laboratories). Five-parameter logistic regression curve (Bio-Plex Manager 6.0) was used to calculate sample concentrations. In addition to previously reported biomarkers (IL-1Ra, MCP1, IL-6, IL-10, MIP1b, and TNF-α) (24), additional markers from the O-Link analysis were MCP2, MCP3, MCP4, CXCL10, OSM, CX3CL1, MCSF, CXCL11, TRAIL, RANKL and IL15 were measured in both Geneva and US cohort. All data below thresholds (last point of the standard curve) were set to half the value of the corresponding threshold.

### ZEBOV-GP-binding antibodies

We used the data generated in studies performed in Geneva, reported in (12) and in the US, reported in (25). For the present study, we refer to measurements performed at day 28 and 180. Briefly, quantification of ZEBOV-GP-specific antibodies for the Geneva cohort was done at the US Army Medical Research Institute for Infectious Diseases (USAMRIID) in Frederick, Maryland, USA in the Diagnostic Systems Division using USAMRIID’s standard operating procedure (SOP AP-03-35; USAMRIID ELISA) (8, 12, 27) by the Filovirus Animal Non-Clinical Group (FANG). For the US cohort, ZEBOV-GP-specific antibodies were tested in Focus Diagnostics, San Juan Capistrano, CA, based on the assay developed by FANG. The homologous Zaire–Kikwit strain GP was used as specified in the SOP. The log10 transformed ELISA units per mL was used for correlation analysis in the present study.

### Identification of the Geneva and US signatures

We applied the same methods as previously (24) to identify signatures of the vaccine response. PCA was done for all participants of each cohort and for all 17 identified markers for which we used the log10 D1/D0 ratio to normalize the data. To build the model, the normalized data were standardized so that the means and the SD equalled 0. PCA components with eigen values greater than 1 were retained. Because of the number of variables introduced in the PCA (n=17) and the number of vaccinees (Geneva cohort: n=100; USA cohort: n=113), a risk of overfitting was suspected, thus a bootstrap procedure was used to check the robustness of the number of retained principal components. For this, 50,000 re-samplings with replacements were done: for each resampling, the same PCA was conducted. Cronbach’s alpha values were used to indicate whether the variation of markers upregulated between days 0 and 1 was based on a single trait. The Kaiser-Meyer-Olkin was used to measure the adequacy of the data to factor analysis (28). Our validation cohort was the US cohort, and we used the same approach to calculate the signature by PCA. The score for each observation was calculated by applying the equations of each component, which then was used to evaluate the correlation with adverse events and biological outcomes.

### Statistical methods

Biomarkers were reported by vaccine dose and timepoint using log10 geometric mean concentrations (GMCs). GMCs were compared between independent groups using t-tests or ANOVA (with Scheffe’s correction for multiplicity of tests and *post hoc* analyses) and over time using linear regression models with mixed effects to account for repeated measures. The association between the signature and biological outcomes/AEs was assessed using linear and logistic regression models with adjustment for the dose. The type I error level was 0.05, and all statistical tests were two-sided. AUCs of the previous and refined signature were compared by using Delong’s non-parametric test for paired ROC curves. Analyses were conducted in R 3.2.2 (R Foundation for Statistical Computing, version 2.15.2) and STATA 14.0 IC (StataCorp LP).

## Results

### Identification of additional biomarkers of innate responses to rVSVΔG-ZEBOV

We set up a pilot experiment using an O-link approach that can measure up to 276 analytes to identify additional plasma markers associated with the vaccine response compared to our previous study (**Figure 1A**). Markers significantly peaked at day 1 in both the high and low dose groups, but not at day 3 or 7 (**Figure 1B**). Therefore, we subsequently only analysed the ratio of D1/D0. In the high-dose (HD) vaccinees group, 18 new additional proteins from the inflammatory panel were significantly elevated and one protein (4EBP1) showed a significant decrease (**Figures 1B, C**). In the low-dose (LD) vaccinees group, 18 new proteins were significantly elevated (16 were shared with HD vaccinees) and one (MMP1) was significantly decreased (**Supplementary Figure 2A**). The analysis of the metabolic and immune panels showed that in the HD group 17 new proteins were significantly increased, and 13 were significantly decreased on day 1 compared to day 0 (**Figure 1D**), whereas in the LD group four new proteins were significantly elevated and eight were significantly decreased (**Supplementary Figure 2B**) (no new markers were shared with HD vaccinees). We observed that all the proteins identified in our previous study (24) had significantly increased on day 1, confirming our previous findings, and supporting the use of O-link as an adequate screening tool. Secreted proteins with a D1/D0 ratio greater than 1 but without statistical significance are shown in **Supplementary Figures 2C, D**. We did not find statistically significant differences in biomarkers levels between arthritis and non-arthritis in this subset of patients in the inflammatory panel and metabolic panel analysed (**Supplementary Table 1**).

In conclusion, use of O-link screening in a subset of the Geneva cohort (n=49) allowed us to identify 18 additional proteins significantly secreted at higher levels on day 1 in both high and low dose groups.

### Confirmation and quantification of the biomarker signature

Out of the 18 additional markers found by O-link, eleven were available for measurement by Luminex and were quantified on days 0, 1, 3, 7 in plasma samples of the entire Geneva cohort (n=115). The eleven markers included chemokines: monocyte chemoattractant protein 2 (MCP2/CCL8), monocyte chemoattractant protein 3 (MCP3/CCL7), monocyte chemoattractant protein 4 (MCP4/CCL13), chemokine C-X3-C motif ligand 1 (CX3CL1/Fractalkine), interferon gamma-induced protein 10 (IP10/CXCL10), interferon-gamma-inducible protein 9 (IP-9/CXCL11); cytokines: Interleukin 15 (IL-15), Oncostatin M (OSM) and macrophage colony-stimulating factor (M-CSF); and ligands: Tumor necrosis factor ligand superfamily member 10 (TRAIL/TNFSF10), Tumor necrosis factor ligand superfamily member 11 (RANKL/TNFSF11).

**Figure 2:**
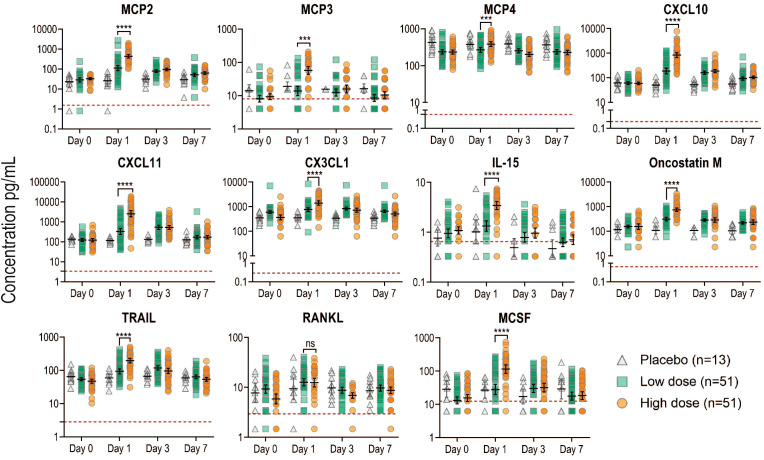
Kinetics of newly identified biomarkers measured in the plasma of all Geneva participants. Plasma concentration in pg/ml for each marker measured by Luminex was plotted at each time point in the different groups: placebo (gray), low dose (green) and high dose (orange). Each dot represents a participant (n=115). Black lines represent the geometric mean concentrations with the CI. Red dotted lines indicate the limit of detection for each marker. Samples below the limit of detection were assigned a value corresponding to 50% of the last standard dilution value. P values less than 0.001 are summarized with three asterisks, and P values less than 0.0001 are summarized with four asterisks.

We calculated the geometric mean concentrations (GMCs) for each marker and the ratio of D1/D0. As expected, in the placebo control group, no marker significantly increased with time, except for CXCL10 that showed a significant decline at day 1 (**Table 1**). We confirmed that all eleven additional markers significantly peaked at day 1 in the Geneva cohort (**Figure 2**), with the largest fold increases reported in HD for CXCL11 [21.0 (95% CI, 15.1 to 29.2)], CXCL10 [14.2 (95% CI, 11 to 18.4)] and MCP2 [13.3 (95% CI, 11 to 16.1)] (**Table 1**). HD vaccinees showed significantly higher increases in GMCs than LD vaccinees for all markers except RANKL (**Figure 2**).

**Table 1:** Ratio day 1/day 0 of the geometric mean (GM) of the additional identified markers measured in the plasma of Geneva participants.

	Placebo (n=13)			Low Dose (n=51)			High Dose (n=51)		
Marker	Ratio GM	Confidence Interval	*p-value*	Ratio GM	Confidence Interval	*p-value*	Ratio GM	Confidence Interval	*p-value*
CXCL11	0,85	(0,68 - 1,07)	0,150	2,65	(1,98 - 3,54)	**<0,001**	21	(15,14 - 29,23)	**<0,001**
CXCL10	0,81	(0,69 - 0,96)	**0,019**	3,08	(2,39 - 3,97)	**<0,001**	14,2	(10,99 - 18,35)	**<0,001**
MCP2	1,12	(0,89 - 1,41)	0,298	3,95	(2,94 - 5,29)	**<0,001**	13,3	(10,95 - 16,14)	**<0,001**
MCSF	0,95	(0,56 - 1,61)	0,831	2,07	(1,58 - 2,72)	**<0,001**	7,41	(5,59 - 9,82)	**<0,001**
MCP3	1,35	(0,83 - 2,22)	0,207	1,71	(1,27 - 2,3)	**<0,001**	6,18	(4,54 - 8,43)	**<0,001**
OSM	0,93	(0,65- 1,33)	0,660	2,01	(1,72 - 2,34)	**<0,001**	4,78	(3,83 - 5,97)	**<0,001**
TRAIL	0,92	(0,76 - 1,12)	0,368	1,72	(1,5 - 1,98)	**<0,001**	4,15	(3,62 -4,76)	**<0,001**
CX3CL1	0,99	(0,77 - 1,28)	0,925	1,27	(1,12 - 1,44)	**<0,001**	3,83	(2,95 - 4,98)	**<0,001**
IL15	1,34	(0,91 - 1,96)	0,123	1,4	(1,19 - 1,65)	**<0,001**	3,15	(2,64 - 3,77)	**<0,001**
RANKL	1,21	(0,74 - 1,96)	0,415	1,36	(1,2 - 1,54)	**<0,001**	2,13	(1,69 - 2,68)	**<0,001**
MCP4	0,9	(0,73- 1,11)	0,295	1,13	(1,06 - 1,21)	**<0,001**	1,64	(1,48 - 1,82)	**<0,001**
IL1-Rα	0,97	(0,78 - 1,21)	0,81	1,77	(1,39 - 2,26)	**<0,001**	10,6	(8,41 - 13,37)	**<0,001**
IL-6	0,7	(0,35 - 1,38)	0,31	1,82	(1,18 - 2,79)	**0,007**	13,5	(8,29 - 21,91)	**<0,001**
IL-10	0,74	(0,39 - 1,38)	0,35	2,11	(1,19 - 3,75)	**0,011**	7,08	(4,68 - 10,70)	**<0,001**
TNF-α	1,3	(0,59 - 2,87)	0,51	1,33	(0,78 - 2,27)	0,3	3,98	(2,43 - 6,51)	**<0,001**
MCP-1	0,89	(0,78 - 1,02)	0,11	1,4	(1,22 - 1,62)	**0,011**	3,35	(2,97 - 3,78)	**<0,001**
MIP-1β	0,96	(0,81 - 1,15)	0,64	1,33	(1,14 -1,55)	**0,011**	2,31	(2,09 - 2,56)	**<0,001**

Ratio of GM: log10 base ratio Day 1/Day 0. Significant difference between day 1 and day 0 are represented by P-values highlighted in bold. Markers are presented according to the ratio GM levels in the high dose. Previous signature biomarkers reported in Huttner et al., 2017 (24) are shaded in grey.

We found that all additional markers except RANKL were significantly correlated between each other and with the previously reported markers, irrespective of the vaccine dose (**Supplementary Figure 3**) The strongest associations were observed between CXCL10 and CXCL11 at both doses (Spearman’s correlation coefficient r = 0.92, p <0.001; r=0.88, p<0.001) and between MCP1 and MCP2 (Spearman’s correlation coefficient r = 0.61, p<0.001; r=0.82, p<0.001 at the two doses respectively) (**Supplementary Figure 3**).

In summary, we found eleven additional markers at day 1 after vaccination that correlated with the previously identified signature in the Geneva cohort.

### Refinement of the innate plasma signature

**Figure 3:**
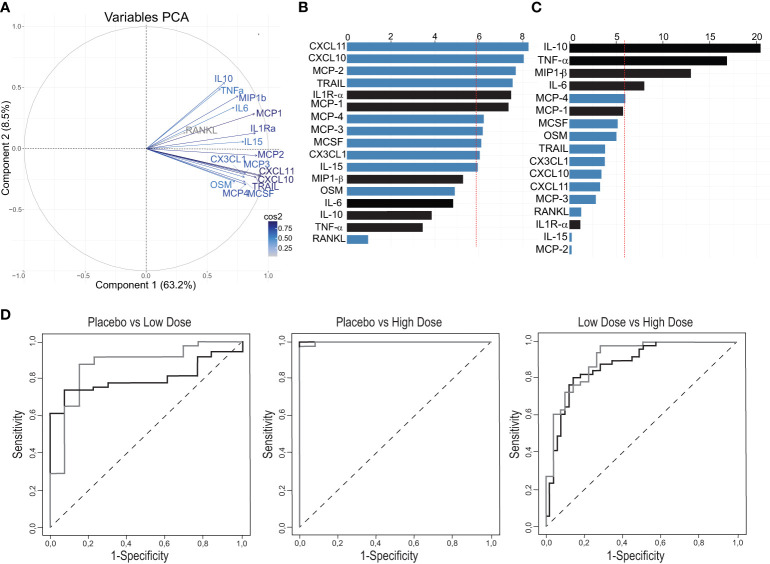
Definition of a refined signature by PCA after rVSVΔG-ZEBOV-GP vaccination in the Geneva cohort. **(A)** A variable correlation plot shows the magnitude (length of the arrow) and direction of the correlations of each marker (n=17) to each of the two principal components. Cos2 values indicate how well represented the marker is on the principal component and are shown in a gradient of colours shown in the legend. **(B, C)** Graphs showing the percentage of the contribution of each marker to the variability on component 1 **(B)** and component 2 **(C)**. Red dashed line indicates the average contribution. Blue bars indicate additional markers and bars in black indicate previous markers. **(D)** Comparison Area Under the Curve (AUC) between previous signature (black line) and refined signature (grey line).

PCA was conducted for the 17 markers described above (6 previously reported and the 11 additional reported here). PCA showed that the new refined signature accounted for 77.8% of the variability of the day 1 immune response versus baseline and three components were retained (PC1: 63.2%, PC2 8.5% and PC3 6.1% of the variance; **Figure 3A**). The bootstrap analysis confirmed the robustness of the first three components. The frequency of the number of retained components (Eigen value > 1) over the 50’000 re-sampling was PC1: n=50000/50000 (100%); PC2: n=49849/50000 (99.7%); PC3: n=34580/50000 (69.16%); PC4: n=113/50000 (0.23%); PC5: n=0/50000 (0%). Cronbach’s alpha values (LD:0.94, HD: 0.94) indicated that the variability in the markers induced by the vaccine was highly reliable and mostly based on a common trait. The overall measure of adequacy was 0.9, considered by Kaiser et al. (28) as very robust data for factor analysis.

After normalization and standardization, the equation of the first component (PC1) was defined by “0.083×IL1Ra^STD^ + 0.067xIL6^STD^ + 0.057xTNFa^STD^ + 0.06xIL10^STD^ + 0.083xMCP1^STD^ + 0.07xMIP1b^STD^ + 0.076xMCP3^STD^ + 0.086xCXCL10^STD^ + 0.068xOSM^STD^ + 0.076xMCP4^STD^ + 0.075xCX3CL1^STD^ + 0.075xMCSF^STD^ + 0.088xCXCL11^STD^ + 0.084xTRAIL^STD^ + 0.084xMCP2^STD^ + 0.03xRANKL^STD^ + 0.074xIL15 ^STD^”, i.e., 17 biomarkers. PC2 equation is reported in **Supplementary Table 2**.

The biomarkers contributing to component 1 were all positively correlated, while the ones contributing to component 2 showed both a positive and negative correlations (**Figure 3A**). In the component 1, eleven biomarkers were above the expected average contribution, six of them strongly contributing to the component variability (CXCL11, CXCL10, MCP-2, TRAIL, IL1Ra, MCP-1; **Figure 3B**), while for the component 2, four biomarkers strongly contributed to component variability (IL-10, TNFA, MP1b, IL-6; **Figure 3C**).

We next found that the refined signature discriminated better than the previous signature between placebo recipients and LD vaccinees [AUC: 0.87 (95% CI, 0.75 to 0.99) vs 0.79 (95% CI, 0.69 to 0.91); p=0.37], and between low- and HD vaccinees [0.91 (95% CI, 0.85 to 0.97) vs 0.88 (95% CI, 0.81 to 0.95); p=0.059]. Both signatures discriminated almost perfectly placebo recipients and HD vaccinees with area under ROC curves close to 1 (**Figure 3D**). Altogether, these results show that the addition of eleven markers refined the previous plasma signature as it explained a higher percentage of the variability in the response and improved the discrimination between the two vaccine doses.

### Additional biomarkers are associated with vaccine-related adverse events

**Figure 4:**
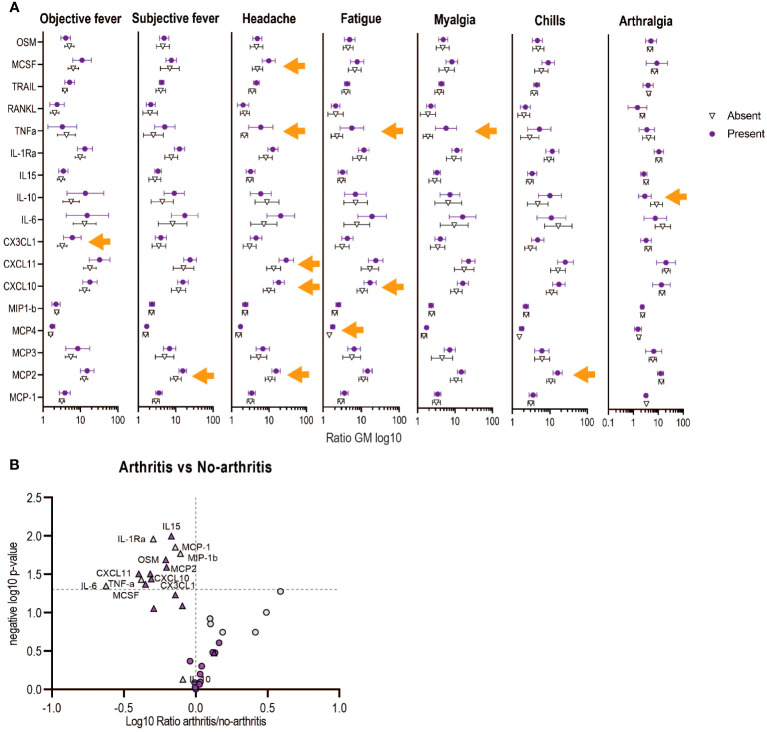
Associations between the refined signature biomarkers with early adverse events (AEs) in Geneva vaccinees receiving high vaccine dose. **(A)** Each symbol represents the ratio of geometric mean (log10 of day 1/day 0) for each biomarker. Bars shown mean and 95% CI. Orange arrows shows significance difference between having or not the indicated AE. **(B)** Volcano plots from the ratio of the plasma markers measured in those with arthritis vs those with no-arthritis in log10 fold change (x axis) against the t-test-derived negative log10 statistical P-value (y axis) for the additional (purple) and previous (grey) biomarkers of the refined signature. Thresholds (dotted grey line): p-value cut-off was fixed at 0.05 (1,3 negative log10) and fold change cut-offs is 1 (0 in the log10 scale). The two vaccine doses are shown with circles (low dose) or triangles (high dose).

We next performed a multivariable analysis to assess whether the refined signature was associated with the risk of adverse events following vaccination, as previously described (24). Similarly, we showed that a score higher than one of the Components 1 and 2 of the refined signature increased the risk of injection-site pain, subjective fever and chills in HD vaccinees, (**Table 2**). In contrast to our previous report, only Component 1 of the refined signature was associated with a higher risk of objective fever and myalgia, while Component 2 was associated with higher risk of headache in HD vaccinees. Because adverse events (AEs) were reported mainly in HD vaccinees (97%), which corresponds to the vaccine dose used in Ervebo^®^, we focused on this group for further analyses. Headache was associated with significant increase in CXCL10, CXCL11, MCSF, MCP-2 and TNF-alpha, while fatigue was associated with significant increases in CXCL10, MCP-4 and TNF-α (**Figure 4A**). Increase in MCP-2 was specifically associated with subjective fever and chills, while CX3CL1 and TNF-α were associated with objective fever and myalgia. In contrast, a significant decrease of the anti-inflammatory cytokine IL-10 was associated with arthralgia. No identified biomarker was associated with local pain. Overall, TNF-α and MCP-2 were key biomarkers associated with most systemic AEs.

**Table 2:** Multivariable analyses of the determinants of clinical outcomes of the refined innate signature in Geneva vaccinees (n=100).

			1st component		2nd component	
Adverse Event	Predictor		Adjusted OR (95%CI)	p-value	Adjusted OR (95%CI)	p-value
Objective fever	Dose	Low dose	Ref		Ref	** **
		High dose	15.99 (2.3 to 331.34)	**0,017**	16.31 (3.03 to 303.15)	**0,009**
	Signature	<0	Ref		Ref	
		>=0	1.05 (0.23 to 5.8)	0,956	0.64 (0.18 to 2.14)	0,472
Subjective fever	Dose	Low dose	Ref		Ref	
		High dose	3.73 (1.36 to 10.78)	**0,012**	5.07 (2.19 to 12.31)	**<0.001**
	Signature	<0	Ref		Ref	
		>=0	1.72 (0.6 to 4.78)	0,302	0.69 (0.29 to 1.61)	0,388
Headache	Dose	Low dose	Ref		Ref	
		High dose	2.14 (0.79 to 5.93)	0,133	2.67 (1.19 to 6.14)	**0,018**
	Signature	<0	Ref		Ref	
		>=0	1.47 (0.53 to 4.01)	0,446	0.63 (0.28 to 1.43)	0,272
Fatigue	Dose	Low dose	Ref		Ref	
		High dose	1.22 (0.43 to 3.58)	0,706	0.75 (0.33 to 1.7)	0,495
	Signature	<0	Ref		Ref	
		>=0	0.44 (0.15 to 1.24)	0,129	1 (0.44 to 2.28)	0,996
Myalgia	Dose	Low dose	Ref		Ref	
		High dose	2.81 (1.04 to 7.98)	**0,045**	3.15 (1.4 to 7.31)	**0,006**
	Signature	<0	Ref		Ref	
		>=0	1.22 (0.43 to 3.31)	0,702	0.61 (0.26 to 1.39)	0,242
Chills	Dose	Low dose	Ref		Ref	
		High dose	3.2 (1.15 to 9.63)	**0,030**	3.1 (1.36 to 7.35)	**0,008**
	Signature	<0	Ref		Ref	
		>=0	0.96 (0.32 to 2.69)	0,935	0.85 (0.37 to 1.95)	0,694
Arthralgia	Dose	Low dose	Ref		Ref	
		High dose	0.97 (0.25 to 3.78)	0,960	1.28 (0.44 to 3.88)	0,655
	Signature	<0	Ref		Ref	
		>=0	1.62 (0.42 to 6.58)	0,486	0.88 (0.3 to 2.6)	0,807
Pain	Dose	Low dose	Ref		Ref	
		High dose	19.64 (5.81 to 91.45)	**<0.001**	17.81 (6.59 to 55.78)	**<0.001**
	Signature	<0	Ref		Ref	
		>=0	0.62 (0.13 to 2.13)	0,484	2.92 (1.06 to 8.98)	0,046

Multivariable analyses were performed to assess the association between the refine innate signature components 1 and 2, and adverse events (AEs) adjusting for the vaccine dose. Logistic regression models were used. The reported adjusted odds ratios (ORs) capture the increase in risk of an AE compared with the reference category (denoted “Ref”). In grey, results that were similar between previous and refined signature.

Significant difference against the reference in the Doses or in the Signature component is represented by P-values highlighted in bold.

Twenty-four percent (24%) of participants reported transient vaccine-induced arthritis in the Geneva cohort (12), which was previously associated with lower day 1 signature scores only in HD vaccinees (24). Here, we report a similar finding, Component 1 was significantly lower in HD vaccinees with transient arthritis (GM non-arthritis 0,93 (0,7-0,17) vs GM arthritis 0,34 (-0,05-0,73) p: 0,011) and levels of seven innate plasma biomarkers were also significantly lower (MCP-2, CXCL10, CXCL11, CX3CL1, MCSF, IL-15, OSM), complementary to the four previous biomarkers reported (IL-6, TNF-a, MCP-1 and MIP-1b) (**Figure 4B**).

Of note, the refined signature showed little to no association with age but was associated with gender (lower scores of Component 1 in females [−0.22 versus 0.19, p=0.029)], confirming what was reported for the previous signature (24).

Overall, the refined signature can thus better predict the risk of objective fever, myalgia and headache and several additional biomarkers were found to be significantly associated with specific systemic adverse events including transient arthritis.

### The refined signature and the additional markers are differentially associated with hematological, virological and immunological outcomes

rVSVΔG-ZEBOV-GP immunization triggers a transient, dose-dependent viremia and hematological changes (8, 12). We observed a significant positive association between Component 1 of the refined signature and viremia mainly in LD vaccinees (**Supplementary Table 3**) that was ruled by IL-15, RANKL and MCSF (**Supplementary Table 4**). We found a negative correlation for both doses between component 1 of the refined signature and day 1 lymphopenia and thrombopenia, which was maintained until day 3 only for HD vaccinees. These negative associations of both doses with lymphopenia were correlated with all additional biomarkers while the negative correlation with thrombopenia was related to different biomarkers (**Supplementary Table 3**). Component 1 was differently associated with neutropenia according to the vaccine dose. Early (day 1) neutropenia was positively associated in HD vaccinees and was influenced mainly by MCP-3, while delayed neutropenia was negatively associated with LD vaccination.

Finally, in this analysis, we found limited correlation of the two PCs with antibody response except for Component 2 in HD vaccinees that positively correlated with antibody levels 180 days after vaccination (**Supplementary Table 3**). Others have reported correlation between the antibody levels at day 28 with day 3 CXCL10 levels when considering all vaccinees irrespective of the vaccine dose (19). A similar univariate analysis grouping the LD and HD groups showed that the antibody levels at day 28 positively correlated with the ratio D1/D0 (or actual concentrations at day 1) of several cytokines and chemokines, including CXCL-10 (**Supplementary Figure 4**). This correlation was limited to a more limited set of cytokines at day 3. Antibody response at day 180 was associated with the D1/D0 ratio of IL-10, MCP-1 and MIP-1b, and in HD only with IL-10 that drives the positive association found with Component 2 in HD vaccinees. In line with the multivariate analysis, there were fewer correlations between the antibody levels at day 28 with innate plasma biomarkers when considering each dose group separately, limited to positive correlation with D1/D0 ratio of MCP-1 and MIP-1b levels (LD group) and negative correlation with CXCL10 level (HD group; **Supplementary Figure 4**).

In summary, component 1 of the refined signature differentially correlated with LD viremia (positive) and hematological (negative) outcomes, several innate plasma biomarkers including CXCL10 were associated with antibody titers one month after vaccination but fewer with long-term specific antibody response.

### Validation of the refined signature in an independent US cohort

The kinetics of the response of the 17 biomarkers in the US cohort was similar to the ones observed in the Geneva participants, although some differences were noted in the magnitude of the response (**Supplementary Figure 5**). In US HD vaccinees, the largest fold increases were observed for IL10 [58.1 (95% CI, 43 to 78)], CXCL10 [57.8 (95% CI, 43 to 79)] and CXL11 [28.6 (95% CI, 20 to 40)]. Although weaker in magnitude, the same markers including MCP-2 showed the largest fold increase in LD vaccinees (**Supplementary Table 5**). At baseline, most biomarkers were significantly lower in the US cohort, while the D1/D0 ratio showed similar responses in both cohorts, CXCL10, CXCL11, IL-10 and MCP-2 being the biomarkers with the highest ratio in both cohorts (**Table 1**; **Supplementary Table 5**).

**Figure 5:**
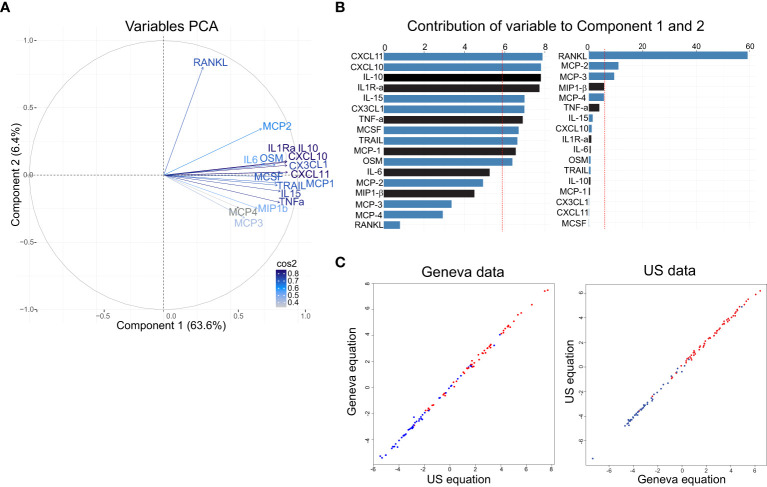
Analysis of the signature in the US cohort and validation of the refine signature defined in the Geneva cohort. **(A)** Variable correlation plot shows the magnitude (length of the arrow) and direction of the correlations of each marker (n=17) to each of the two principal components. Cos2 values indicates how well represented the marker is on the principal component and are shown in a gradient of colours: grey represent low values, light blue represents mid values, dark blue represents high values. **(B)** Percentage of contribution to the variability of each marker in the component 1 (left) and component 2 (right). Red dashed line indicates the expected average contribution. Bars in blue indicate additional markers and in black previous markers **(C)** Correlation between Geneva equation and US equation using Geneva data (left) and US data (right).

To evaluate whether the signature defined using the Geneva cohort could predict rVSVΔG-ZEBOV-GP responses elicited in a different cohort, we applied an independent PCA to the US data. Similar to what was found in Geneva, three components explained 75.9% of the variability of the D1/D0 ratios (PC1 explained 63.6% of the variance, PC2: 6.4% and PC3: 5.9%) (**Figure 5A**). The bootstrap showed that the first three components were robust (PC1: n=50000/50000 (100%), PC2: n=49333/50000 (98.67%), PC3: n=27657/50000 (55.31%). The overall measure of adequacy was 0.93. Thus, the PCA model in the US samples was adequate and behaved very similarly as for the Geneva samples.

Comparable to what was observed in Geneva cohort, the first component also discriminates well between LD and HD (**Supplementary Figure 6**) and had a similar equation for component 1: “0.085×IL1Ra^STD^ + 0.07xIL6^STD^ + 0.08xTNFa^STD^ + 0.085xIL10^STD^ + 0.078xMCP1^STD^ + 0.064xMIP1b^STD^ + 0.056xMCP3^STD^ + 0.085xCXCL10^STD^ + 0.077xOSM^STD^ + 0.052xMCP4^STD^ + 0.08xCX3CL1^STD^ + 0.079xMCSF^STD^ + 0.085xCXCL11^STD^ + 0.078xTRAIL^STD^ + 0.067xMCP2^STD^ + 0.027xRANKL^STD^ + 0.08xIL15 ^STD^”. Component 2’s equation is shown in **Supplementary Table 2**. In addition, biomarkers contributing to component 1 were positively correlated, while the ones contributing to component 2 had both a positive and negative correlations (**Figure 5A**). In component 1, eleven biomarkers were above the expected average contribution with CXCL11, CXCL10, IL-10 and ILR-α being the highest, while for the component 2, three biomarkers contributed to the component variability, with RANKL representing 58% of the contribution (**Figure 5B**).

We next asked whether applying the Geneva first two components to the US data and vice versa would generate comparable results. Only the first component correlated strongly in both data sets, using Geneva data (r = 0.97, P < 0.001) and using US data (r = 0.99, P=0) (**Figure 5C**), and discriminated well the participants receiving the LD and the HD in both cohorts (**Supplementary Figure 6**).

The validation confirms that Component 1 of the refined signature accurately predicts the variability in response to the rVSVΔG-ZEBOV-GP vaccine.

### GP-specific antibody levels also correlate with biomarkers in the US cohort

Similar to Geneva cohort, when considering all vaccinees irrespective of the vaccine dose the antibody levels at day 28 in all vaccinees positively correlated with the D1/D0 ratio of several cytokines, such as IL1RA, IL-10, MCP-1, CXCL10, MIP1b, CX3CL1, MCSF, CXCL11, TRAIL, IL-15. The correlation was also mostly lost when considering day 3 cytokine ratio and when splitting by dose (**Supplementary Figure 4**). Unlike in the Geneva cohort, most of these correlations were maintained until day 180 after vaccination (**Supplementary Figure 4**).

Overall, US cohort innate plasma signature biomarkers also correlate with antibody levels at day 28 and 180 after vaccination with rVSVΔG-ZEBOV-GP.

## Discussion

We showed that the inclusion of additional biomarkers refined the first plasma signature identified previously in Geneva. The refined signature, which now includes 17 markers, better discriminated between vaccine doses as it performed better at capturing the variability of the vaccine responses, and better defined the risk of fever, myalgia and headache. We also found new biomarkers that correlated with reactogenicity and transient arthritis, and that were associated with antibody levels one and six months after vaccination. Finally, the results were cross validated in a separate cohort.

We used O-link to screen for additional markers: of the many markers screened, only 18 were significantly higher in both HD and LD vaccinees. These markers are related to monocytes recruitment as well as to biological processes involved in vaccine responses such as pro-inflammatory cytokines, chemokine-signaling pathways, chemotaxis of different immune populations (monocytes, neutrophils, eosinophils and lymphocytes) and cellular response to interferon gamma. CXCL10, CXCL11, MCP-2, IL1R-α were the markers with the highest D1/D0 ratio as well as the ones with the greatest contribution to the variability of Component 1. CXCL10 and CXCL11 are IFN-dependent cytokines and plays an important role in the chemotaxis of monocytes, T-cells, NK cells and dendritic cells. They are secreted by monocytes, endothelial cells and fibroblasts, and their secretion is enhanced in the presence of TNF-α (29). This is in line with the positive correlation that we observed between CXCL10 and CXCL11 with TNF-α. Previous transcriptomic analysis from blood samples of the same cohorts have shown that interferon signaling genes (ISGs) were upregulated at day 1 post-vaccination and, consistent with our results, CXCL10 was upregulated at day 1 (30, 31). Similarly, the replication incompetent Ebola vaccine Ad26.ZEBOV increases the expression of IFN-stimulated genes (CXCL9, CXCL11, and CXCL10), and those associated with monocyte and lymphocyte recruitment such as CCL2 (MCP-1),CCL8 (MCP-2), and CCL7 (MCP-3) (32). However, compared to rVSVΔG-ZEBOV-GP, Ad26-ZEBOV combined with MVA-BN-Filo (Zabdeno/Mvabea) as well as another adenovirus-based Ebola vaccine cAd3-EBOZ is less immunogenic with less persisting antibody response, requiring higher doses to reach the same level of immunogenicity (33, 34).

Of note, we did not detect an increase in plasma IFN protein level (similar to previous reports (19)) but CXCL10 and CXCL11 increase may result from a transient and earlier IFN response before day 1. This discrepancy between gene expression and the protein level of IFN in blood might reflect rapid migration of cells to secondary lymphoid organs (33), rapid kinetics of the IFNs secretion (32) and/or a sub-optimal sensitivity of the assay used to detect these proteins. The innate vaccine response induced by the live attenuated rVSVΔG-ZEBOV-GP it is mainly related to monocyte recruitment and activation, whereas live-attenuated yellow fever mainly induces a dendritic-cell (DC) innate signature (35, 36) and the adjuvanted influenza-H1N vaccine induces a lymphoid gene-expression signature (37). More recently, SARSCoV-2 infection as well as mRNA vaccination were shown to induce a monocyte and DC innate signature with enhanced serum levels of IFN-α (38) and IFN-gamma, respectively (39).

Compared with the first signature reported previously (24), the refined signature presented herein explains a higher proportion of the variability of the D1/D0 ratios. Components 1 and 2 were both very robust and included different biomarkers that contributed to their variability, which can explain the different associations observed with dose, adverse events and biological outcomes. For instance, in contrast with the previous signature, component 1 was associated with risk of objective fever and myalgia, while component 2 (which represented only 8.5% of the variability) was the only one significantly associated with a risk of headache and with the GP-specific antibody response six months after vaccination.

Another important distinction with the previous signature was that several specific biomarkers were associated with the presence of systemic adverse events in HD vaccinees. Most of these associations were with single markers, for example high levels of CX3CL1 and MCP-2 were associated with the presence of objective fever and subjective fever, respectively. Increase in CX3CL1 plasma level has been associated with Hanta virus fever (40). CX3CL1 shedding can be induced by MCP-1 via p38 signaling (41). This is in line with the positive correlation we saw between plasma levels of CX3CL1 and MCP-1, suggesting that MCP-1 could induce shedding of CX3CL1. In addition, the correlation between fatigue and headache with TNF-α plasma levels found in the previous signature is now extended to several additional biomarkers including CXCL10.

Similarly, the risk of transient arthritis after vaccination is associated with the reduction of various additional biomarkers mainly in HD vaccinees. After rVSVΔG-ZEBOV-GP vaccination, 24% of Geneva trial reported transient arthritis and the virus was isolated in the synovial fluid (12). While in US trial the frequency of reported transient arthritis was 5%, the cases were dispersed across multiple doses including placebo (7, 9), likely confounding a direct comparison. In agreement with the previous signature (24), we found in Geneva cohort that Component 1 was significantly lower in HD vaccines who developed arthritis, this was ruled by 12 out of 17 biomarkers constituting the signature that had significantly lower plasma levels in HD vaccinees with arthritis. The topmost differentially expressed markers were IL-6, CXCL10, CXCL11, TNF-α and MCSF. Although the roles of IL-6 and TNF-α in rheumatoid arthritis (42, 43) and in chronic chikungunya arthritis (44) are well established, we saw a reduction during the acute phase. However, it is also well established that a robust cytokine response during the acute phase of viral infection is vital for clearance and control of viral dissemination, and prevention of chronic chikungunya arthritis (45). Our results suggest that individuals who developed arthritis after a HD vaccine (which in close to the dose currently in use in the field 72x10^6^pfu/dose)had a lower level of inflammatory response and therefore, we hypothesize have a less effective early control of viral dissemination, which may in turn leads to viral presence in privileged sites such as joints, and thus could enhance the risk of vaccine-induced viral arthritis (8, 12, 46). The lack of association with bone resorption markers such as RANKL (47) is in line with the absence of bone resorption lesions in our arthritis patients (12), in contrast to chikungunya arthritis (46). Recently, transcriptomic analysis of the same Geneva cohort identified an early five-gene signature associated with the risk of arthritis that included T-cell subset genes CD4 and CCR7, IFN-regulatory sign gene FCGR1A, myeloid-associated gene IL12A, and Th2-associated gene GATA3 (30). Taken together, we hypothesized that the loss of T-cell homeostasis, a weak innate response during the acute phase (in HD vaccinees) and age at the time of vaccination (in LD vaccinees) are associated with transient arthritis after rVSVΔG-ZEBOV-GP vaccination.

We did not analyze the impact of baseline in the incidence of the adverse events observed after vaccination, but this was evaluated using machine learning in other paper by members of the consortium using the same cohorts as in the present study. In this study, 22 genes at baseline were associated with fatigue, headache, myalgia, fever, chills, arthralgia, nausea and arthritis (48).

Others have reported a correlation between the early innate response and specific-GP antibody levels one month after rVSVΔG-ZEBOV-GP vaccination that involved upregulation of ISGs such as IFI6 gene at day 7 (30) and CXCL10 protein levels at day 3 (19). We also found a positive correlation in both cohorts between specific-GP antibody titers one month after vaccination in all vaccinees and D1/D0 ratio of several innate plasma signature biomarkers including CXCL10 and IL-15. IL-15 and IFN-g have been reported to correlate with antibody response after the second dose of BTN162b2 mRNA COVID-19 vaccine (49). We also saw that in US cohort more innate biomarkers correlate with the antibody levels compared to the Geneva cohort; we can not exclude that this could be due to a difference in the vaccine dose in the two countrioes, since for the HD groups participants in the US received 100x10^6^pfu/dose (n=30) and 20x10^6^pfu/mL (n=30), while in Geneva, participants received 10x10^6^pfu/dose (n=35) 50x10^6^pfu/dose (n=16).These results highlight the key role of early activation of interferon-dependent responses at the transcriptional and protein level in the generation of high antibody levels, as reported for other vaccines (49–53).

We validated this refined signature in a US cohort. Although baseline levels of IL-10 were higher in the US than in the Geneva cohort, the kinetics of the biomarkers as well as the components of rVSVΔG-ZEBOV-GP early response were remarkably comparable. This implies that innate responses induced after rVSVΔG-ZEBOV-GP vaccination were very robust, likely independent of genetic and environmental background. The biomarkers that contributed to the US Component 1 variability were similar to the ones in Geneva’s, except for IL-10, which was significantly higher for the US Component 1. In contrast, Component 2 in the two cohorts have different sets of markers that contribute to the variability. For instance, in Geneva IL-10, TNF-a, MIP-1b and IL-6 are the main contributors, whereas for the US cohort the main contributors are RANKL, MCP-2 and MCP-3.

The study identified certain markers by O-link, with CLEC4G/4C/4D/6A showing significant increases in the high-dose (HD) group, while only CLEC4C increased in the low-dose (LD) group. These markers belong to C-type lectin ligands receptors (CLRs), recognized as pattern recognition receptors (PRRs) and are crucial for initiating innate immune responses. CLEC4G, known as LSECtin, serves as an attachment factor for Ebola and SARS viruses, (54) and plays an important role in Ebola GP-mediated inflammatory responses in human DCs by inducing TNF-α and IL-6 secretion (55). CLEC4C is found exclusively on plasmacytoid dendritic cells (pDCs) and can bind various cells and viruses, including HIV-1 and hepatitis C virus (56, 57, Florentin et al., 2011). CLEC6A (Dectin-2) is an FcRγ-coupled receptor on macrophages and dendritic cells, proposed as a potential attachment factor for Ebola (56). CLEC4D (MCL) is a macrophage C-type lectin implicated in the upregulation of innate genes post-vaccination. Altogether, this suggest that CLEC proteins that increased after rVSVΔG-ZEBOV-GP vaccination may have the potential to bind to the Ebola glycoprotein. This binding could lead to the activation of monocytes, macrophages, and dendritic cells. However, further research is required to fully understand the role of these CLEC proteins in the context of vaccination.Our study has limitations, we were not able to quantify all the markers that were found with the initial O-link screening because they are not available within the Luminex technology, a technique that we had to use to allow comparison with our previous study. Binding antibody responses were assessed at different labs on samples from two different cohorts. This may have also led to some variability in correlation analysis. It would also be interesting to conduct *in vitro* studies to define which cells produce these biomarkers associated with AEs upon rVSVΔG-ZEBOV-GP exposure, in particular cells from the joint, skin or vascular.

In conclusion, we refined the early plasma innate signature induced by rVSVΔG-ZEBOV-GP vaccine, which now better correlates with the presence of AEs, hematological changes, viremia and antibody titer in Geneva cohort. This refined signature was validated in an independent US cohort and showed strong correlation between cohorts, demonstrating its robustness and potential for broad applicability. This innate refined plasma signature highlights the importance of the innate response, especially of monocytes, in the development of rVSV-vaccine responses, and its potential role in controlling vaccine dissemination to prevent arthritis. Altogether, these results provide new insights into early blood biomarkers of immunogenicity and reactogenicity of the rVSVΔG-ZEBOV-GP vaccine.

## Data availability statement

The raw data supporting the conclusions of this article will be made available by the authors, without undue reservation.

## Ethics statement

The study involved humans and were approved by Ethics committees of the WHO for both cohorts. Local Ethics committees for Geneva trial: Canton of Geneva and and Swiss Agency for Therapeutic Products (Swissmedic). Local committees for USA trial: the Chesapeake Institutional Review Boards (Columbia, MD, USA) and the Crescent City Institutional Review Board (New Orleans, LA, USA). The studies were conducted in accordance with the local legislation and institutional requirements. The human samples used in this study were acquired from previous studies for which ethical approval was obtained as mentioned before and informed consent for participation was signed. For this study extra written informed consent for participation was not required from the participants or the participants’ legal guardians/next of kin in accordance with the national legislation and institutional requirements.

## Author contributions

PM-M: Conceptualization, Investigation, Methodology, Project administration, Validation, Visualization, Writing – original draft, Writing – review & editing. AH: Conceptualization, Writing – review & editing, Supervision. SL: Data curation, Formal analysis, Methodology, Visualization, Writing – review & editing. DM: Conceptualization, Funding acquisition, Resources, Writing – review & editing. TO: Conceptualization, Funding acquisition, Resources, Writing – review & editing. AH: Conceptualization, Funding acquisition, Resources, Writing – review & editing. AD: Supervision, Visualization, Writing – original draft, Writing – review & editing. C-AS: Conceptualization, Funding acquisition, Methodology, Project administration, Resources, Supervision, Writing – original draft, Writing – review & editing.

## Group members of VEBCON Consortium

*Gabon* Selidji Todagbe Agnandji, Sanjeev Krishna, Peter G. Kremsner, Jessica S. Brosnahan (Centre de Recherches Médicales de Lambaréné). *Germany* Selidji Todagbe Agnandji, Sanjeev Krishna, Peter G. Kremsner, Jessica S. Brosnahan (Institut für Tropenmedizin, Universitätsklinikum Tübingen); Marylyn M. Addo (University Medical Center Hamburg); Stephan Becker, Verena Krähling (Institute of Virology, Marburg). *UK* Sanjeev Krishna (St. George’s University of London). *Kenya* Philip Bejon, Patricia Njuguna (Kenya Medical Research Institute, Kilifi). S*witzerland* Claire-Anne Siegrist, Angela Huttner (Geneva University Hospitals, Geneva); and Marie-Paule Kieny, Vasee Moorthy, Patricia Fast, Barbara Savarese, Olivier Lapujade (World Health Organization, Geneva).

## Group members of VSV-EBOVAC Consortium

Selidji Todagbe Agnandji, Rafi Ahmed, Sravya S. Nakka, Floriane Auderset, Philip Bejon, Luisa Borgianni, Jessica Brosnahan, Simone Lucchesi, Olivier Engler, Mariëlle C. Haks, Ali M. Harandi, Donald Gray Heppner, Alice Gerlini, Angela Huttner, Peter Gottfried Kremsner, Donata Medaglini, Thomas Monath, Francis Ndungu, Patricia Njuguna, Tom H. M. Ottenhoff, David Pejoski, Mark Page, Gianni Pozzi, Francesco Santoro, Claire-Anne Siegrist.

## Group members of VSV-EBOPLUS Consortium

Selidji Todagbe Agnandji, Sravya S. Nakka, Luisa Borgianni, Annalisa Ciabattini, Sheri Dubey, Michael Eichberg, Olivier Engler, Patrícia Gonzalez-Dias, Peter Gottfried Kremsner, Ali M. Harandi, Alice Gerlini, Angela Huttner, Donata Medaglini, Helder Nakaya, Tom H. M. Ottenhoff, Gianni Pozzi, Sylvia Rothenberger, Francesco Santoro, Eleonora Vianello, Claire-Anne Siegrist.
