# Supplementary material for: Refined innate plasma signature after rVSVΔG-ZEBOV-GP immunization is shared among adult cohorts in Europe and North America
Source: Front Immunol. 2024 Jan 3;14:1279003. doi: 10.3389/fimmu.2023.1279003 (PMC10791923; doi:10.3389/fimmu.2023.1279003)
Supplement: Supplementary file 1 [file Presentation_1.pptx]

## Slide 1
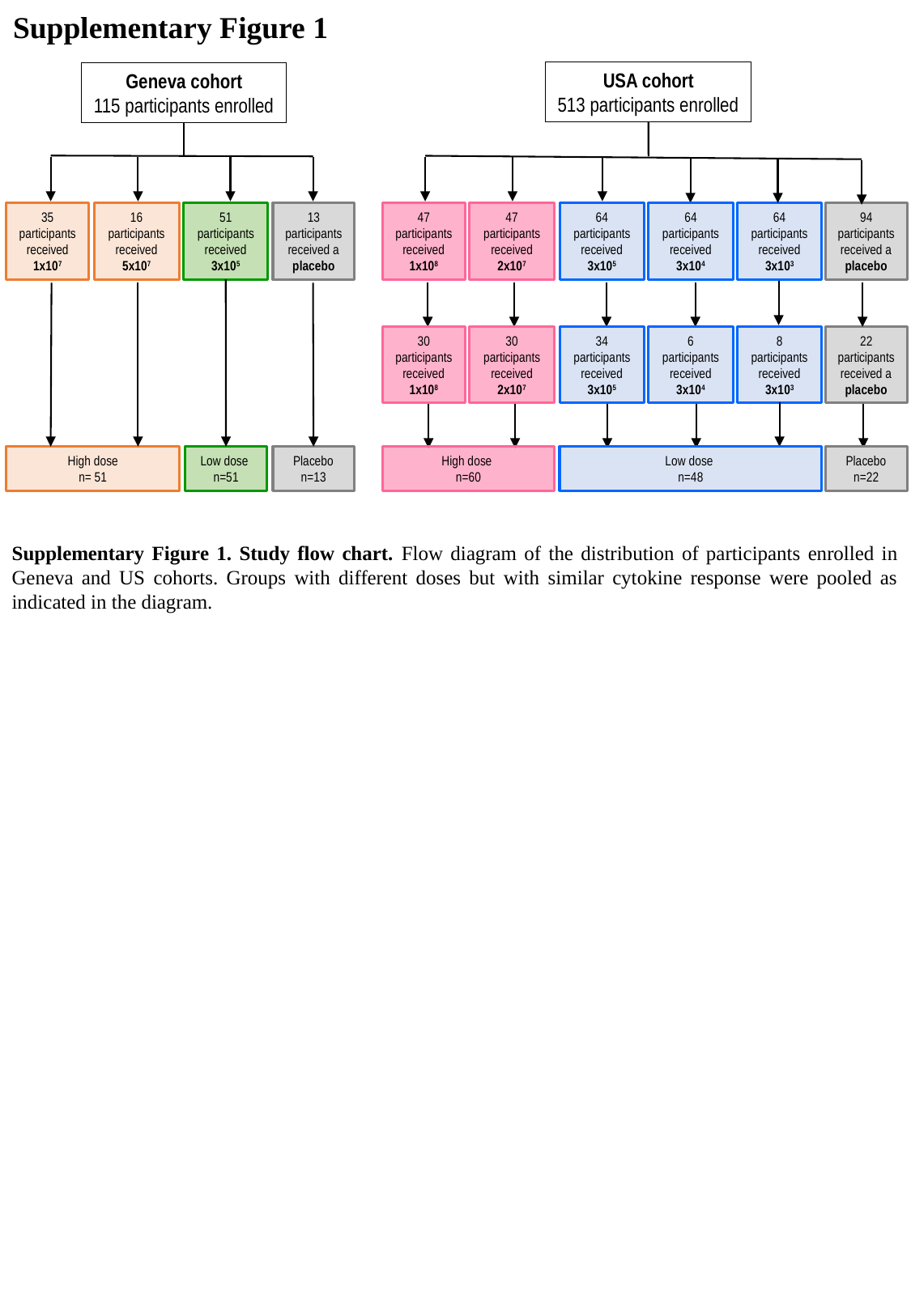

Supplementary Figure 1
USA cohort
513 participants enrolled
Geneva cohort
115 participants enrolled
35 participants received 1x10^7^
16 participants received 5x10^7^
51 participants received 3x10^5^
13
participants received a placebo
47 participants received 1x10^8^
47 participants received 2x10^7^
64 participants received 3x10^5^
64 participants received 3x10^4^
64 participants received 3x10^3^
94
participants received a placebo
34 participants received 3x10^5^
6 participants received 3x10^4^
8 participants received 3x10^3^
22
participants received a placebo
30 participants received 1x10^8^
30 participants received 2x10^7^
High dose
n= 51
Low dose
n=51
Placebo
n=13
High dose
n=60
Low dose
n=48
Placebo
n=22
Supplementary Figure 1. Study flow chart. Flow diagram of the distribution of participants enrolled in Geneva and US cohorts. Groups with different doses but with similar cytokine response were pooled as indicated in the diagram.

## Slide 2
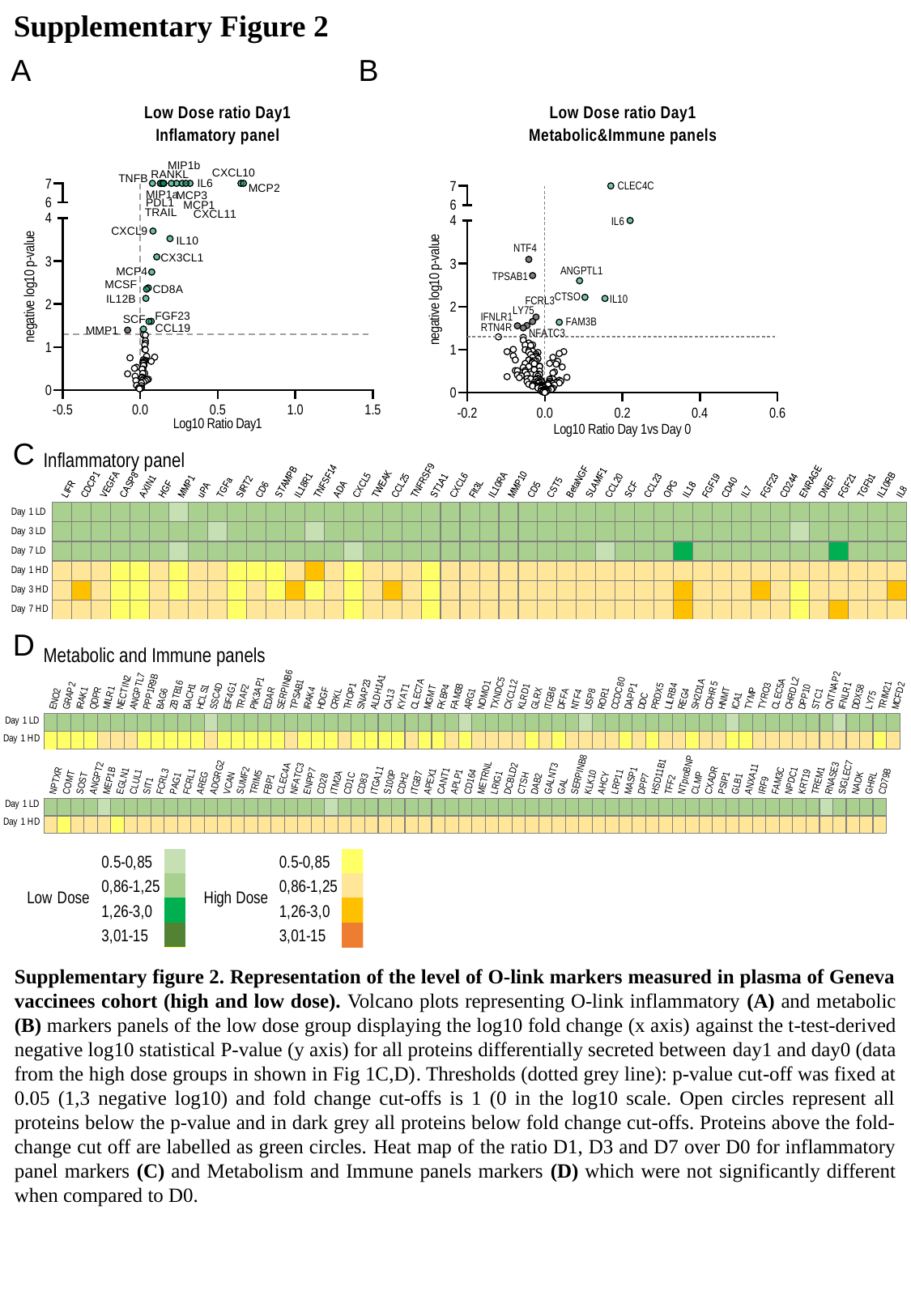

Supplementary Figure 2
A
B
C
Inflammatory panel
D
Metabolic and Immune panels
Supplementary figure 2. Representation of the level of O-link markers measured in plasma of Geneva vaccinees cohort (high and low dose). Volcano plots representing O-link inflammatory (A) and metabolic (B) markers panels of the low dose group displaying the log10 fold change (x axis) against the t-test-derived negative log10 statistical P-value (y axis) for all proteins differentially secreted between day1 and day0 (data from the high dose groups in shown in Fig 1C,D). Thresholds (dotted grey line): p-value cut-off was fixed at 0.05 (1,3 negative log10) and fold change cut-offs is 1 (0 in the log10 scale. Open circles represent all proteins below the p-value and in dark grey all proteins below fold change cut-offs. Proteins above the fold-change cut off are labelled as green circles. Heat map of the ratio D1, D3 and D7 over D0 for inflammatory panel markers (C) and Metabolism and Immune panels markers (D) which were not significantly different when compared to D0.

## Slide 3
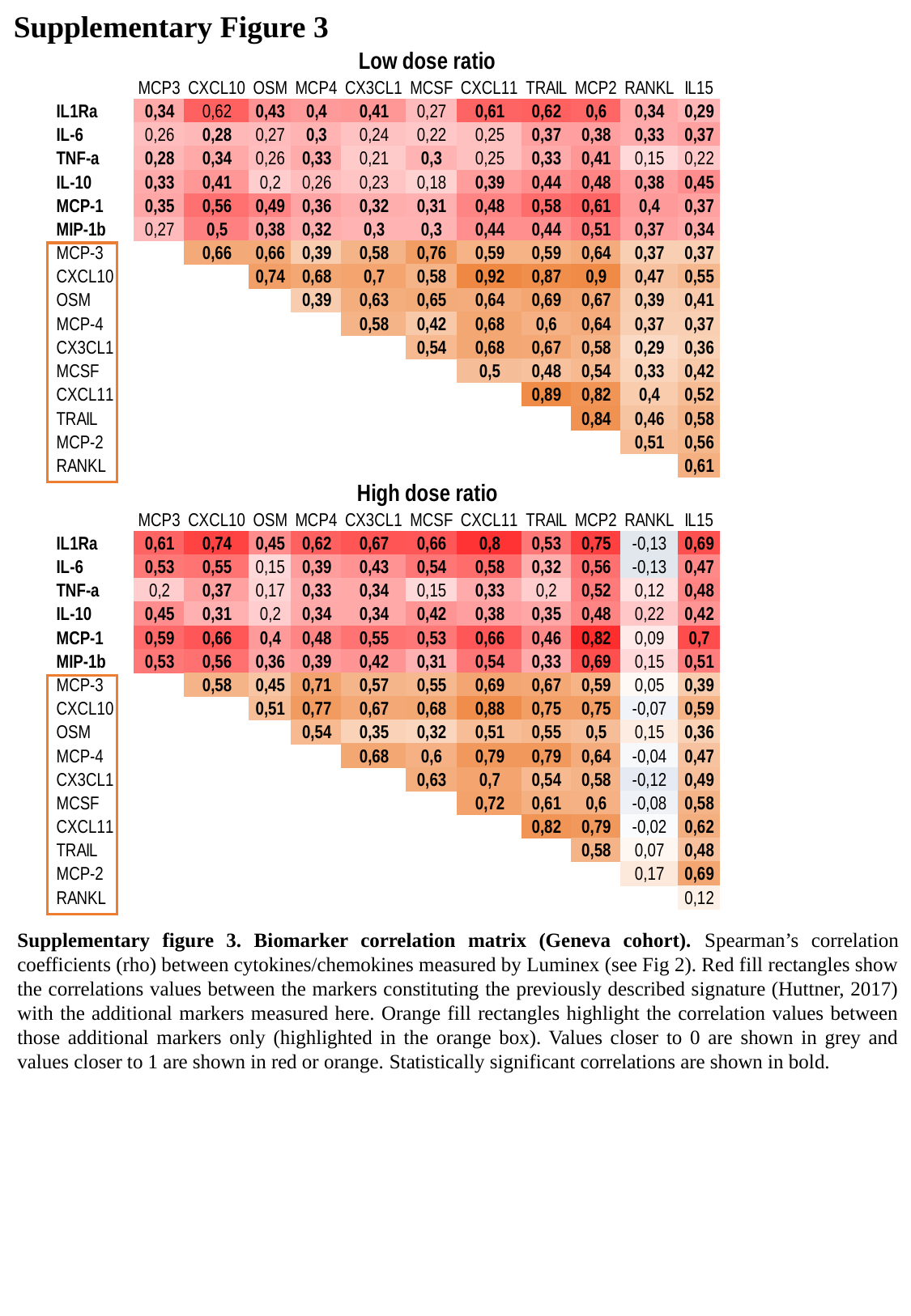

Supplementary Figure 3
Supplementary figure 3. Biomarker correlation matrix (Geneva cohort). Spearman’s correlation coefficients (rho) between cytokines/chemokines measured by Luminex (see Fig 2). Red fill rectangles show the correlations values between the markers constituting the previously described signature (Huttner, 2017) with the additional markers measured here. Orange fill rectangles highlight the correlation values between those additional markers only (highlighted in the orange box). Values closer to 0 are shown in grey and values closer to 1 are shown in red or orange. Statistically significant correlations are shown in bold.

## Slide 4
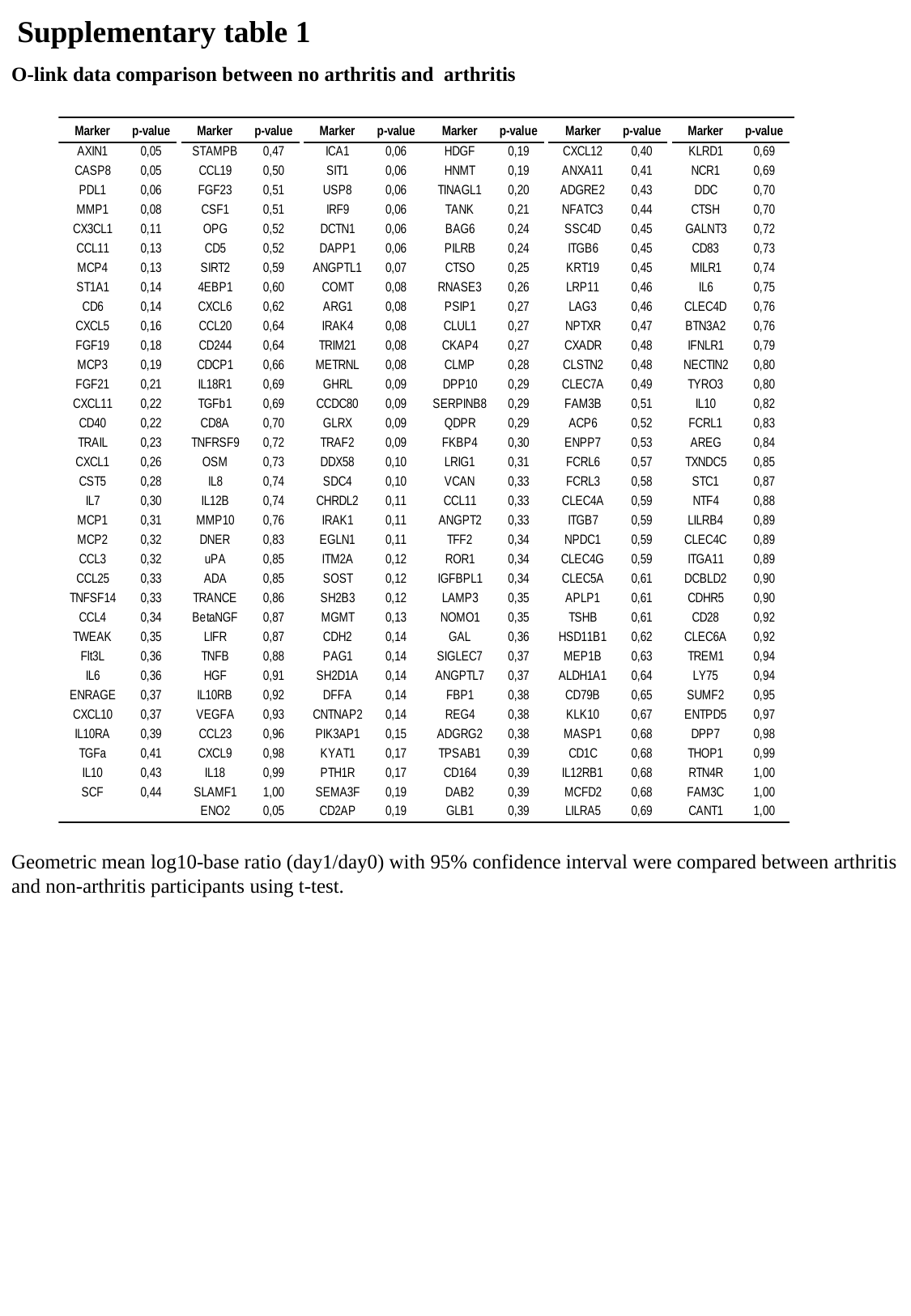

Supplementary table 1
O-link data comparison between no arthritis and arthritis
Geometric mean log10-base ratio (day1/day0) with 95% confidence interval were compared between arthritis and non-arthritis participants using t-test.

## Slide 5
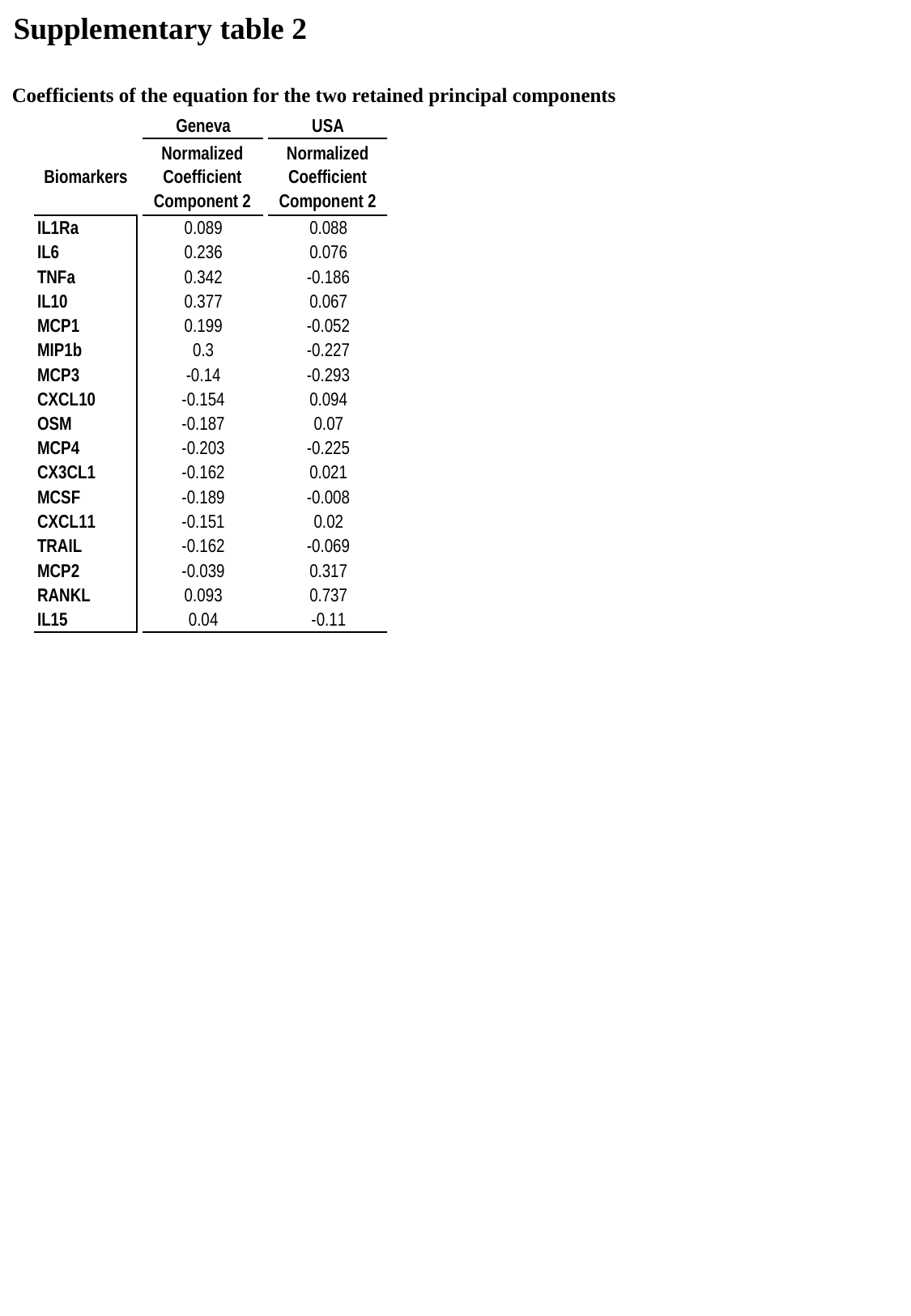

Supplementary table 2
Coefficients of the equation for the two retained principal components

## Slide 6
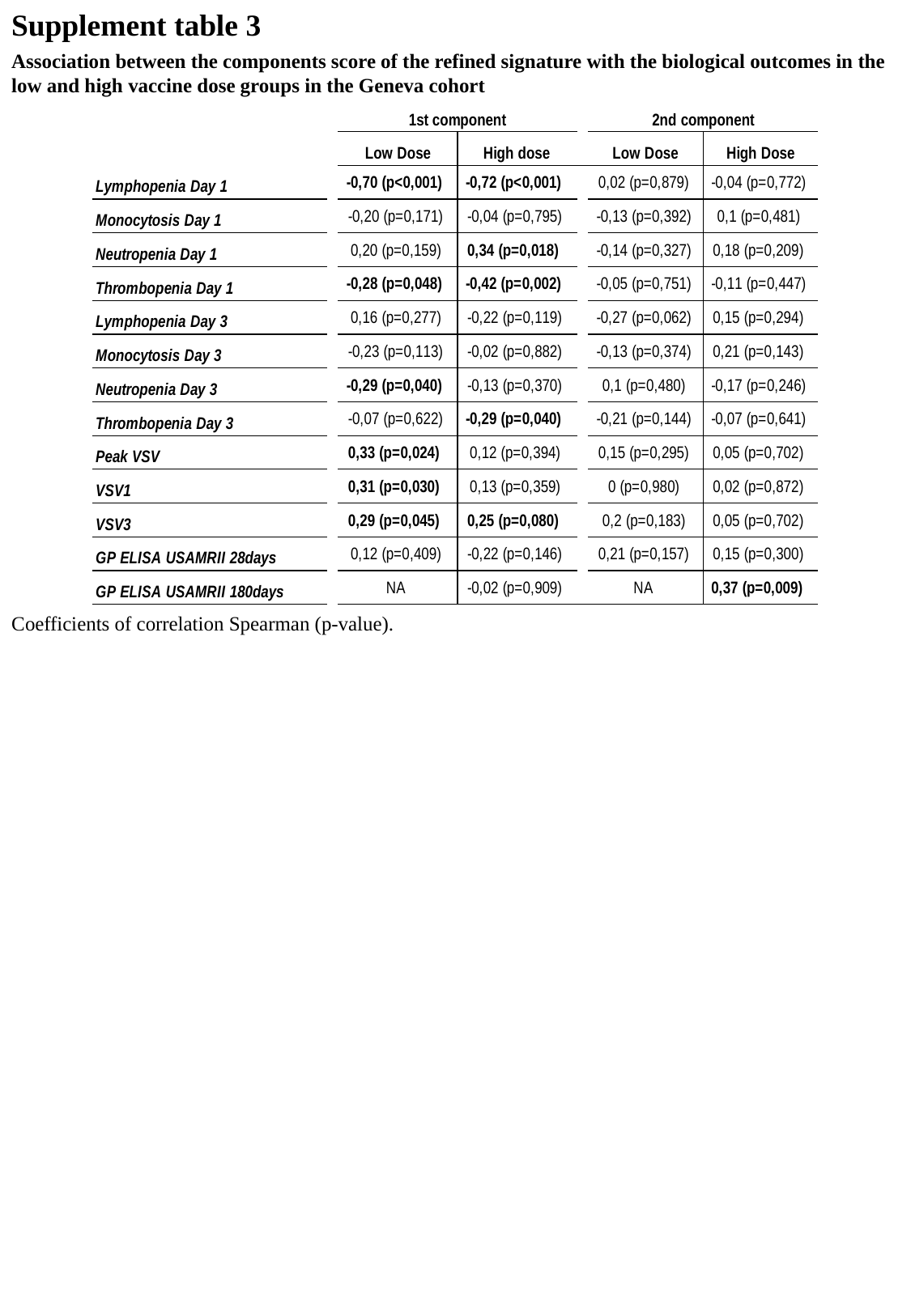

Supplement table 3
Association between the components score of the refined signature with the biological outcomes in the low and high vaccine dose groups in the Geneva cohort
Coefficients of correlation Spearman (p-value).

## Slide 7
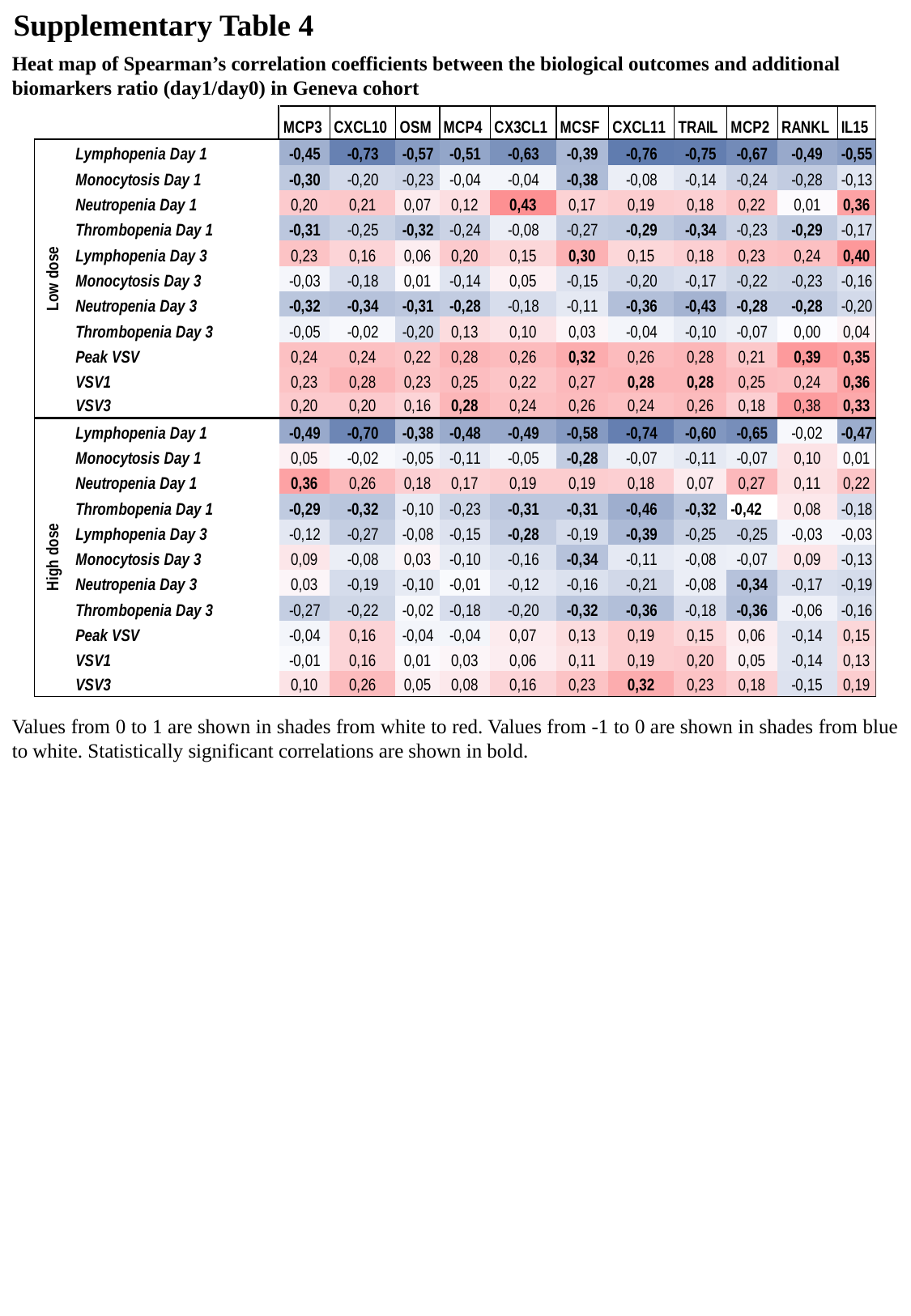

Supplementary Table 4
Heat map of Spearman’s correlation coefficients between the biological outcomes and additional biomarkers ratio (day1/day0) in Geneva cohort
Values from 0 to 1 are shown in shades from white to red. Values from -1 to 0 are shown in shades from blue to white. Statistically significant correlations are shown in bold.

## Slide 8
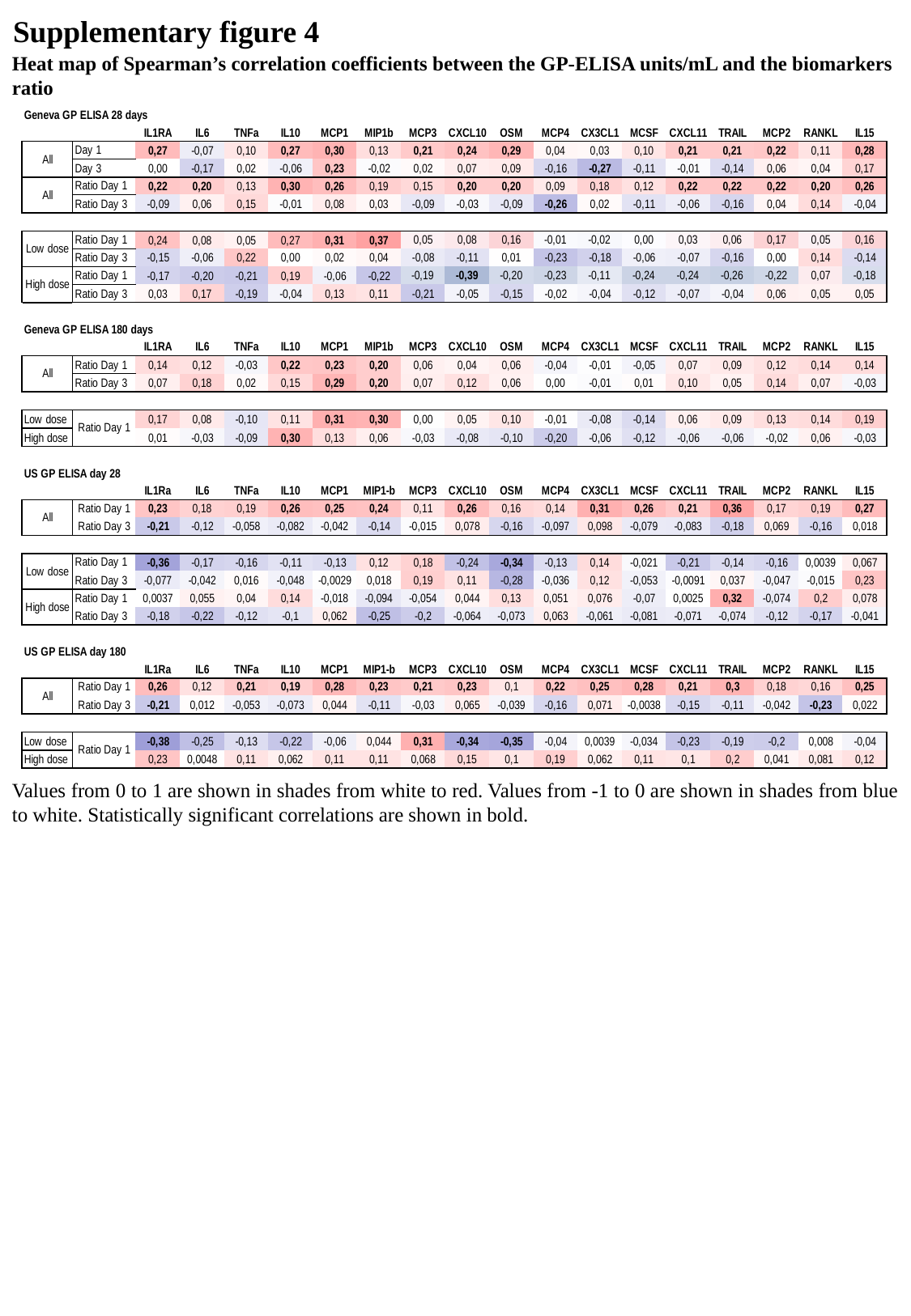

Supplementary figure 4
Heat map of Spearman’s correlation coefficients between the GP-ELISA units/mL and the biomarkers ratio
Values from 0 to 1 are shown in shades from white to red. Values from -1 to 0 are shown in shades from blue to white. Statistically significant correlations are shown in bold.

## Slide 9
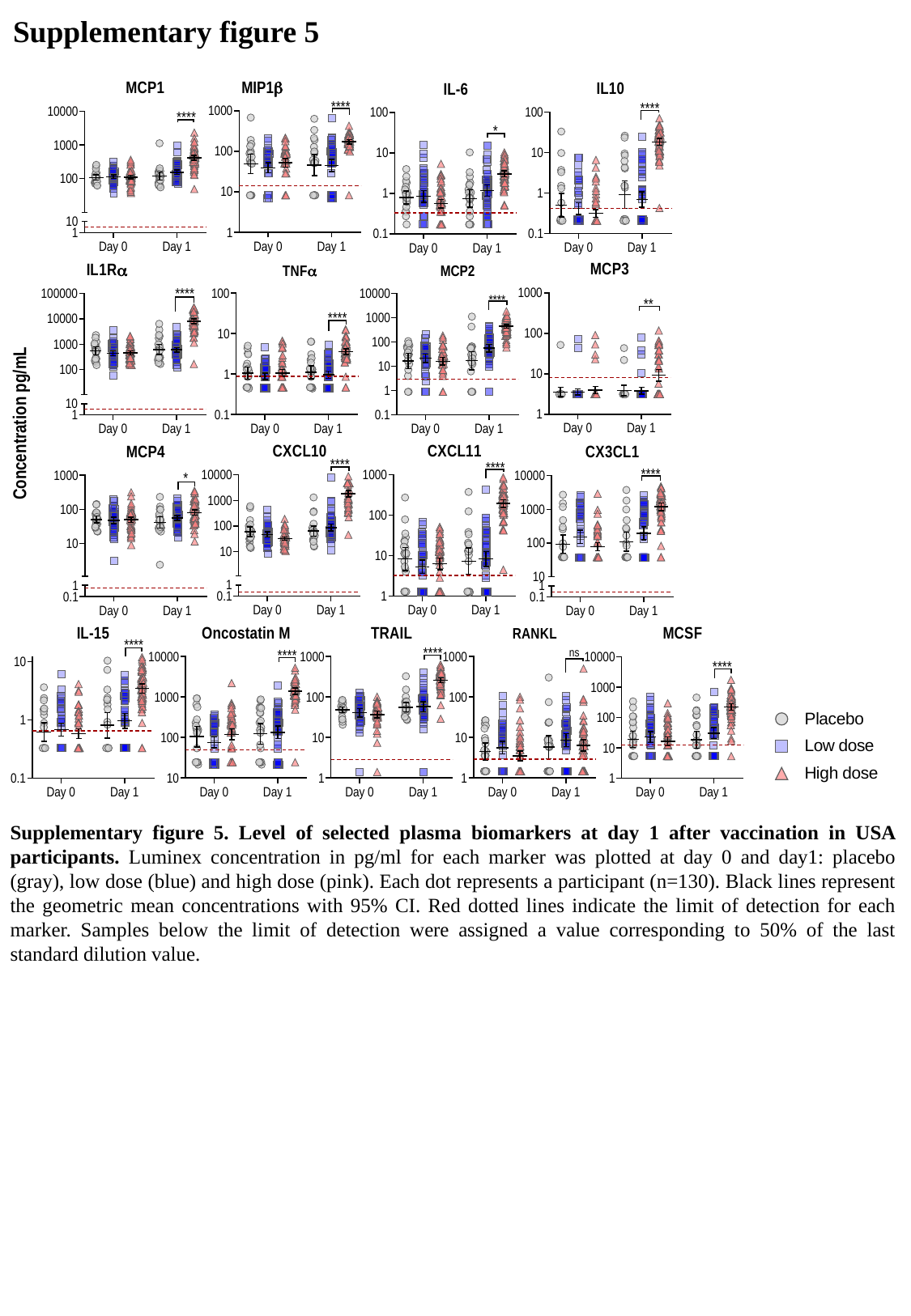

Supplementary figure 5
Concentration pg/mL
Supplementary figure 5. Level of selected plasma biomarkers at day 1 after vaccination in USA participants. Luminex concentration in pg/ml for each marker was plotted at day 0 and day1: placebo (gray), low dose (blue) and high dose (pink). Each dot represents a participant (n=130). Black lines represent the geometric mean concentrations with 95% CI. Red dotted lines indicate the limit of detection for each marker. Samples below the limit of detection were assigned a value corresponding to 50% of the last standard dilution value.

## Slide 10
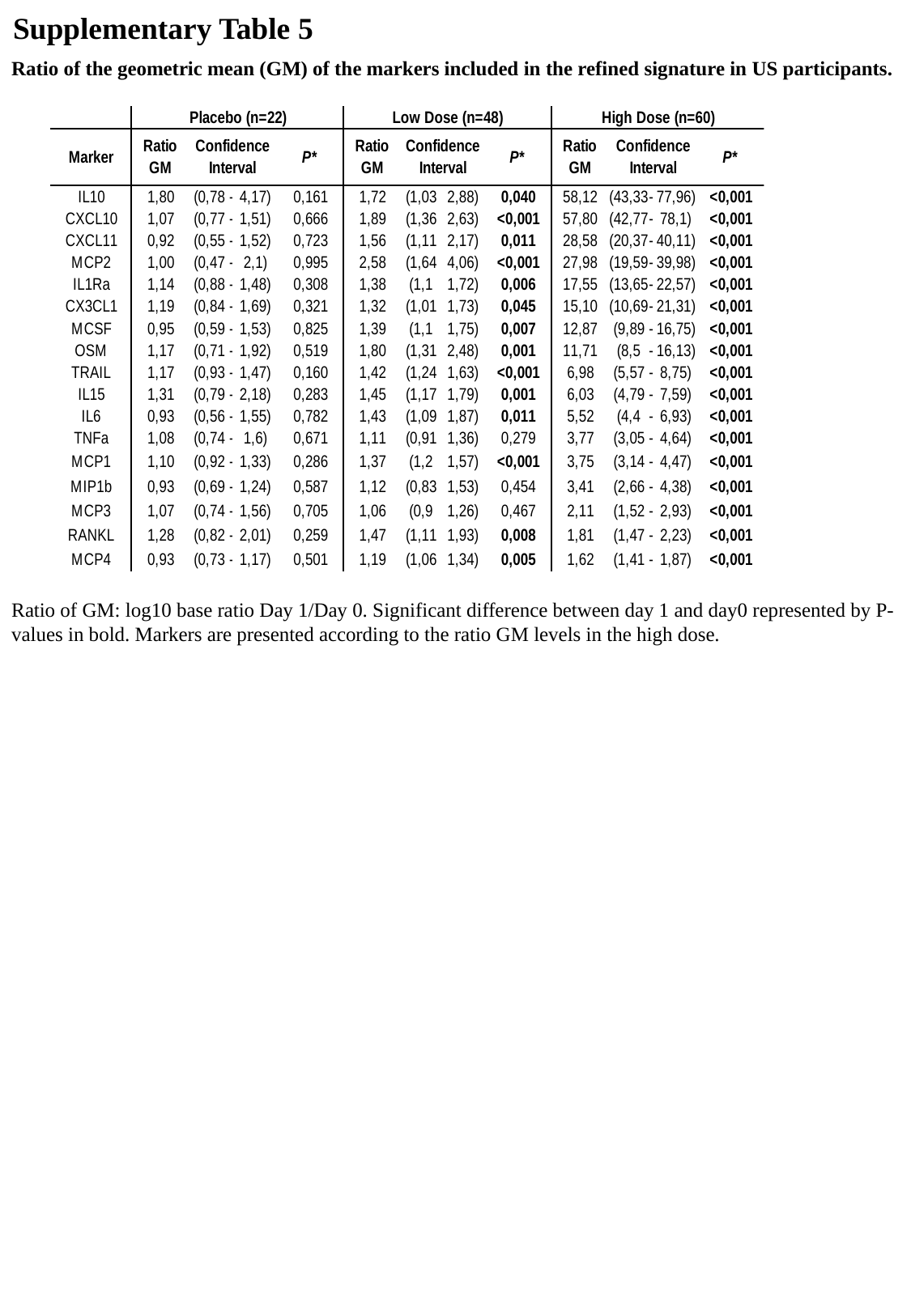

Supplementary Table 5
Ratio of the geometric mean (GM) of the markers included in the refined signature in US participants.
Ratio of GM: log10 base ratio Day 1/Day 0. Significant difference between day 1 and day0 represented by P-values in bold. Markers are presented according to the ratio GM levels in the high dose.

## Slide 11
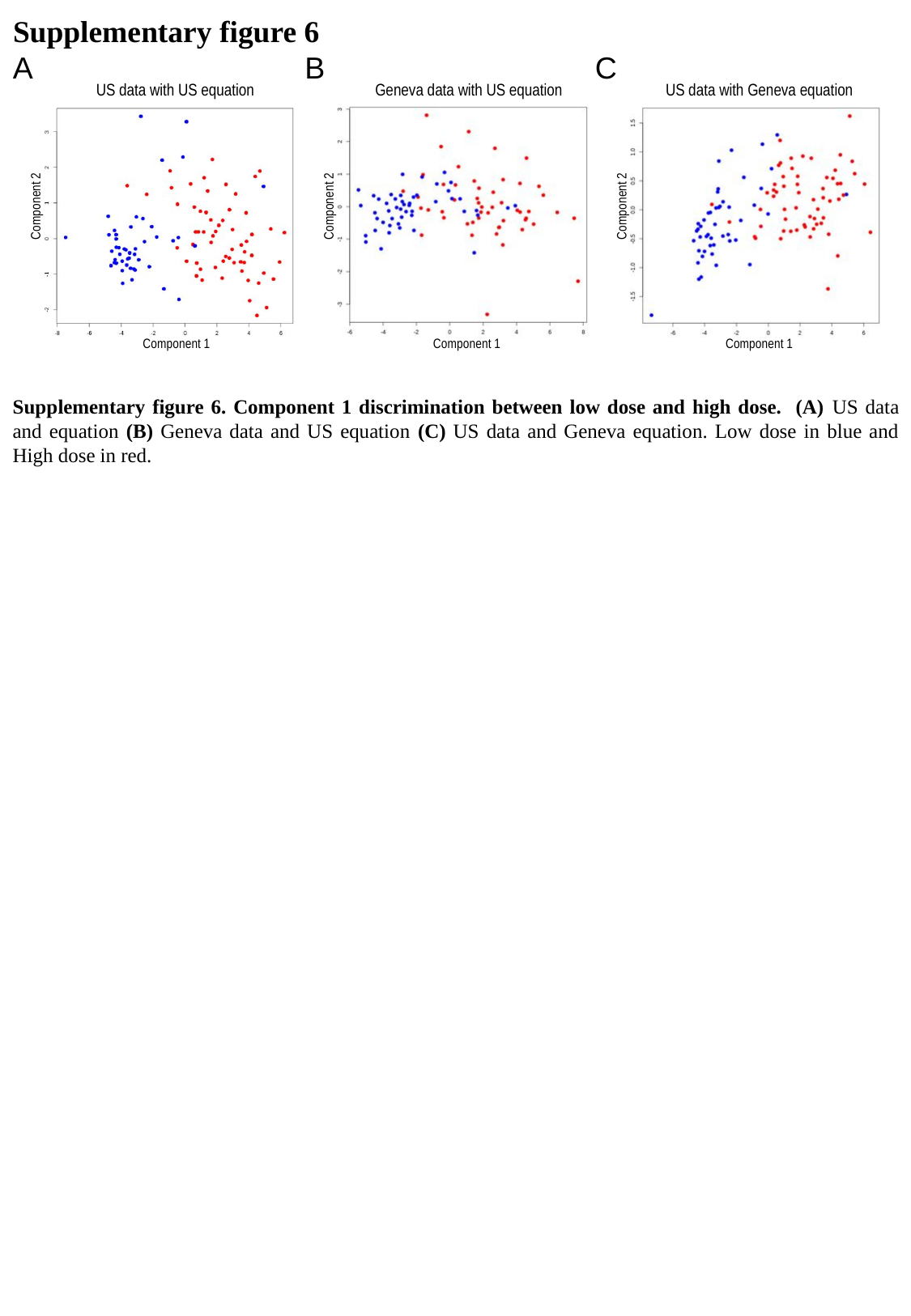

Supplementary figure 6
A
B
C
US data with US equation
Geneva data with US equation
US data with Geneva equation
Component 2
Component 2
Component 2
Component 1
Component 1
Component 1
Supplementary figure 6. Component 1 discrimination between low dose and high dose. (A) US data and equation (B) Geneva data and US equation (C) US data and Geneva equation. Low dose in blue and High dose in red.
